# An All‐Solid‐State Rechargeable Chloride Ion Battery

**DOI:** 10.1002/advs.201802130

**Published:** 2019-01-28

**Authors:** Chao Chen, Tingting Yu, Meng Yang, Xiangyu Zhao, Xiaodong Shen

**Affiliations:** ^1^ College of Materials Science and Engineering Jiangsu Collaborative Innovation Center for Advanced Inorganic Functional Composites Nanjing Tech University Nanjing 211816 China; ^2^ State Key Laboratory of Materials‐Oriented Chemical Engineering Nanjing Tech University Nanjing 211816 China

**Keywords:** chloride ion batteries, electrochemistry, iron oxychloride, polymer electrolytes, rechargeable batteries

## Abstract

The chloride ion battery has been developed as one of the alternative battery chemistries beyond lithium ion, toward abundant material resources and high energy density. Its application, however, is limited by the dissolution of electrode materials and side reactions in the liquid electrolyte. Herein, a solid polymer electrolyte allowing chloride ion transfer and consisting of poly(ethylene oxide) as the polymer matrix, tributylmethylammonium chloride as the chloride salt, and succinonitrile as the solid plasticizer is reported. The as‐prepared polymer electrolyte shows conductivities of 10^−5^–10^−4^ S cm^−1^ in the temperature range of 298–343 K. When it is assembled with the iron oxychloride/lithium electrode system, reversible electrochemical redox reactions of FeOCl/FeO at the cathode side and Li/LiCl at the anode side are realized, demonstrating the first all‐solid‐state rechargeable chloride ion battery.

## 1 Introduction

With the increasing shortage of fossil energy and the pollution of the environment, the development of sustainable clean energy and its harvest, conversion, storage, and usage have been focused.^1^, ^2^, ^3^ Rechargeable batteries have been considered as one of the typical energy storage technologies for different applications in portable electronics, mobile instruments, and stationary grid‐scale stations.^4^ Besides the commercial batteries such as lead‐acid batteries,^5^ Ni‐MH batteries,^6^ and lithium ion batteries,^7^ various alternative battery chemistries based on cation (Na^+^, K^+^, Mg^2+^, Ca^2+^, Zn^2+^, Al^3+^),^8^, ^9^, ^10^, ^11^, ^12^, ^13^ anion (F^−^, Cl^−^, O^2−^),^14^, ^15^, ^16^, ^17^, ^18^ or dual ion (Li^+^/TFSI^−^, AlCl_4_^−^/Al^3+^, Ca^2+^/PF_6_^−^, Li^+^/Mg^2+^; TFSI = bis(trifluoromethylsulfonyl)imide) transfer have been increasingly studied.^19^, ^20^, ^21^, ^22^ With regard to anion battery systems, the chloride ion battery (CIB) is intriguing owing to that the nontoxic and abundant chloride‐containing materials for both electrode and electrolyte are available worldwide.^23^ Moreover, the CIB shows a variety of potential electrochemical couples with high theoretical volumetric energy density up to 2500 Wh l^−1^, which is higher than those of conventional lithium ion batteries.^24^

The concept of CIB was first realized by employing the Lewis‐acid metal chloride as cathode, the alkali or alkaline metal as anode, and the binary ionic liquids as electrolyte, in which the chloride ionic liquid was dissolved in the other ionic liquid solvent with the similar organic cation and another anion such as BF_4_^−^ or TFSI^−^. However, the dissolution and shuttle of metal chloride cathodes in this liquid electrolyte led to severe capacity decay of the CIB.^16^ An alternative approach using metal oxychloride materials such as BiOCl and FeOCl or chloride ion–doped conducting polymer materials with high stability as cathodes has been performed to circumvent this dissolution issue.^25^, ^26^, ^27^, ^28^, ^29^ Nevertheless, these new cathode materials show a reduced energy density as compared with the metal chloride cathodes. For the anode materials, lithium metal with good compatibility with the liquid electrolyte was used as anode of CIBs, in order to investigate the cathode materials. Note that a significant advantage of CIBs is the possible use of abundant materials such as Na, Mg, and Ca as anodes.^30^ The feasibility of Mg anode in CIBs has been proved by employing the metal oxychloride/Mg or metal oxychloride/Mg‐MgCl_2_ electrode systems, which showed poor electrochemical properties that were inferior to those of the electrode systems using lithium metal anode, although the magnesium composite anode materials were used.^30^, ^31^ This inferior performance may be caused by the dissolution of the discharged product of MgCl_2_ at the anode side in the liquid electrolyte, and also the side reactions between magnesium metal and large TFSI^−^ anion of the solvent in the liquid electrolyte,^31^, ^32^ which could be decomposed on the surface of magnesium metal and blocking the magnesium during cycling. In addition, the impurities in the binary ionic liquid electrolyte may also deteriorate the magnesium anode.

One possible solution to dissolve the problems concerning electrode dissolution and side reactions between electrode material and solvent/liquid electrolyte is the use of solid electrolyte, which has been proved to be very effective in lithium ion batteries and metal–sulfur batteries.^33^, ^34^, ^35^ Nevertheless, there are only a few works regarding the solid chloride ion conductors. The inorganic chloride ion conductors such as BaCl_2_, SrCl_2_, LaCl_3_, and LaOCl polycrystalline materials with relatively high electrochemical stability require a high temperature of above 500 K to achieve an ionic conductivity of about 10^−6^ S cm^−1^.^36^, ^37^ Some other binary metal chlorides such as PbCl_2_‐based materials possess a high ionic conductivity of above 10^−5^ S cm^−1^ at room temperature,^38^, ^39^ but their electrochemical stability is low. A higher ionic conductivity of 10^−4^ S cm^−1^ at room temperature has been obtained for the ternary cubic CsSnCl_3_ material, as one of the perovskite‐type chlorides.^39^, ^40^ However, the use of CsSnCl_3_ as the electrolyte is not possible due to its semiconducting feature.^40^ Organic chloride ion conductors, which are based on quasi‐solid‐state polymer‐based materials, have also been reported. Hardy and Shriver developed a poly(diallyldimethylammonium chloride) (PDDAC) plasticized with a liquid poly(ethylene glycol) (PEG) as the chloride ion–conducting polymer electrolyte with a conductivity of 10^−5^ S cm^−1^ at room temperature,^41^ which could be increased to 10^−4^ S cm^−1^ by adding a quaternary ammonium chloride salt.^42^ Similar concept has also been performed to prepare the quasi‐solid‐state polymer electrolyte composed of a polymer matrix of the polyvinyl chloride, gelatin, or polyvinyldifluoride‐hex‐afluoropolymer, a quaternary ammonium chloride salt and a liquid plasticizer of glycerol, PEG, or di‐*n*‐pentylphthalate.^43^ This kind of electrolyte was employed to construct batteries; however, the evidence of the electrochemical reactions based on chloride ion transfer is weak. The as‐assembled batteries with high water content may cause severe chemical chloride ion corrosion. Furthermore, the rechargeability of the as‐assembled batteries is not provided and only the first discharge behavior was shown. Herein, we report the first all‐solid‐state rechargeable chloride ion battery (ASS‐RCIB) that uses a polyethylene oxide (PEO)‐based material as a solid polymer electrolyte (SPE), an iron oxychloride material as a cathode, and lithium metal as an anode. The structure, morphology, and conductivity of the SPE have been analyzed depending on its composition. The battery performance and the electrochemical reaction mechanism related to chloride ion transfer have been investigated.

## 2 Results and Discussion

**Figure 1:**
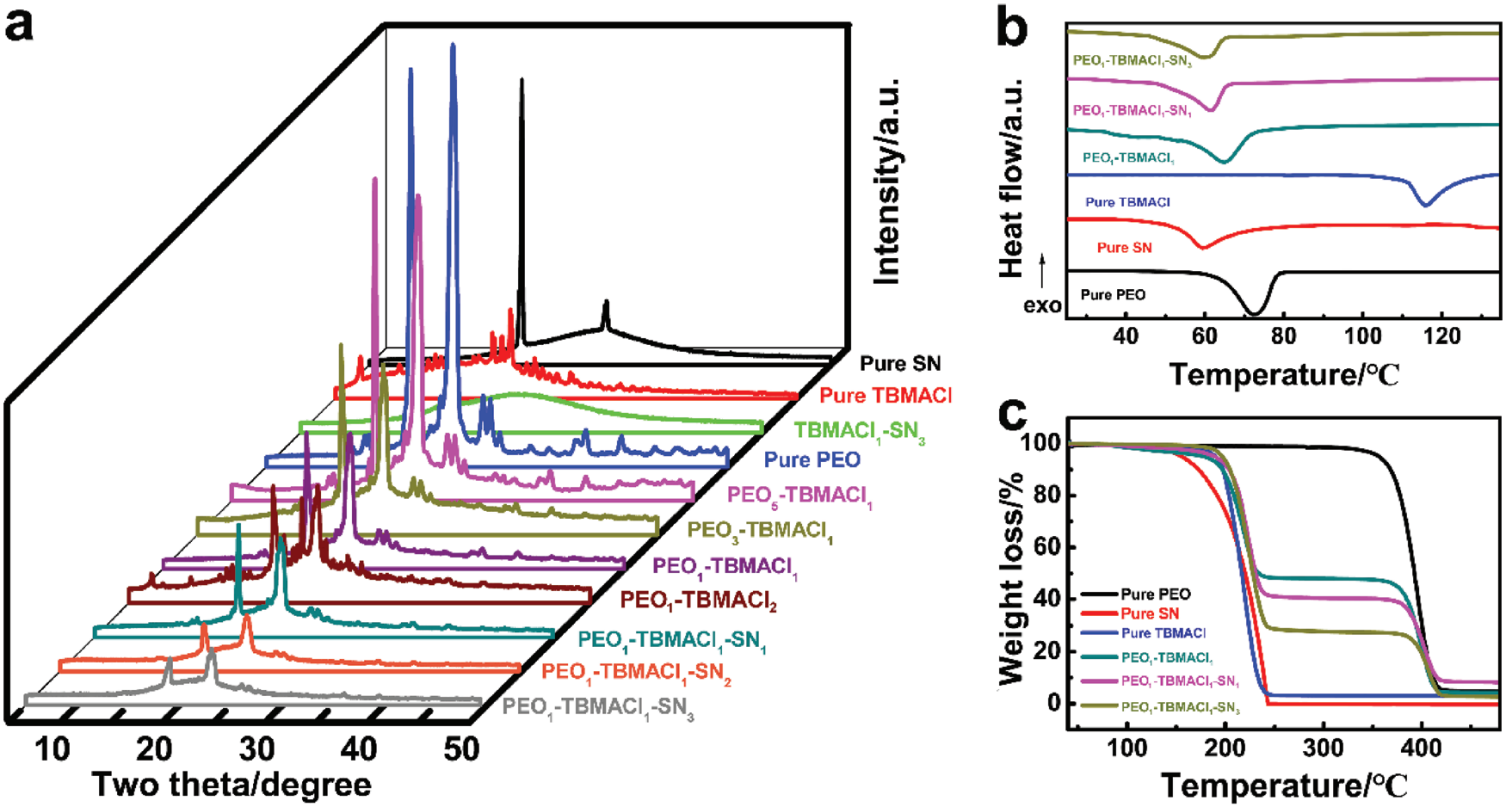
a) XRD patterns of the pure PEO, SN and TBMACl, binary SPE films of PEO‐TBMACl and SN‐TBMACl, and ternary SPE films of PEO‐TBMACl‐SN. b) DSC and c) TGA curves of the pure PEO film, pure SN, pure TBMACl, and SPE films of PEO_1_‐TBMACl_1_, PEO_1_‐TBMACl_1_‐SN_1_, and PEO_1_‐TBMACl_1_‐SN_3_ at a heating rate of 10 °C min^−1^.

**Figure** **1**a shows the X‐ray diffraction (XRD) patterns of the pure PEO, succinonitrile (SN) and tributylmethylammonium chloride (TBMACl), binary SPE films of PEO‐TBMACl and SN‐TBMACl, and ternary PEO‐TBMACl‐SN SPE films. The pure PEO sample has diffraction peaks that are identical to those previously reported,^44^ indicating its monoclinic crystalline structure. The pure SN sample shows a plastic crystal structure with both amorphous and crystalline phases, as reported in literature.^45^, ^46^ The crystalline species exhibits two sharp characteristic peaks at 20.0° and 28.0° corresponding to (110) and (200) lattice planes, respectively. The TBMACl salt possesses a crystalline structure with dozens of diffraction peaks. The broad background centered at about 20.0° was ascribed to the use of amorphous 3M tape for preventing the chloride salt from moisture absorption during the testing. When the TBMACl was mixed with SN in a 1:3 mass ratio, the diffraction peaks of each component almost disappeared, and instead, an evident amorphous structure was formed, indicating the strong chemical interaction between TBMACl and SN due to the high polarity of SN. For the binary PEO‐TBMACl system, the TBMACl can be fully dissolved in the PEO matrix when the mass ratio of PEO and TBMACl is above 1:1, as confirmed by the disappearance of the TBMACl diffraction peaks. Excess TBMACl would be separated from the PEO matrix as the mass ratio decreases to 1:2 (PEO_1_‐TBMACl_2_). The addition of SN in the binary PEO‐TBMACl system contributes to a further decrease in crystallinity, as indicated by the evidently weakened and broadened diffraction peaks.

Differential scanning calorimetry (DSC) analysis, as shown in Figure 1b, was performed to further understand the crystallinity of the pure powders and SPEs. The addition of TBMACl in the PEO matrix leads to a decrease of the melting enthalpy from 174.0 (PEO) to 134.4 J g^−1^ (PEO_1_‐TBMACl_1_), indicating a decrease of the crystallinity, which was reduced continuously with the subsequent introduction of SN. Much lower melting enthalpies of 103.2 and 84.2 J g^−1^ were obtained for the PEO_1_‐TBMACl_1_‐SN_1_ and PEO_1_‐TBMACl_1_‐SN_3_ SPEs, respectively. Similar variation tendency of the melting point (i.e., the midpoint of the endothermic peak), which was reduced from 72.6 (PEO) to 59.3 °C (PEO_1_‐TBMACl_1_‐SN_3_), was also observed. Figure 1c shows the corresponding thermogravimetric analysis (TGA). The PEO matrix possesses good thermal stability up to a high temperature of 340 °C, which was enhanced to 370 °C with the addition of 50 wt% TBMACl (PEO_1_‐TBMACl_1_). The TBMACl exhibits similar thermal stability of about 180 °C in both the pure sample and the binary PEO_1_‐TBMACl_1_ SPE. The pure SN has the lowest thermal stability and it began to lose weight at about 120 °C, which is similar to that in the previous report.^45^ Note that the ternary PEO‐TBMACl‐SN SPE started a drastic weight loss at about 180 °C. This demonstrates that the thermal stability of SN was improved by the chemical interaction in the composite SPE, which would be further analyzed later.

**Figure 2:**
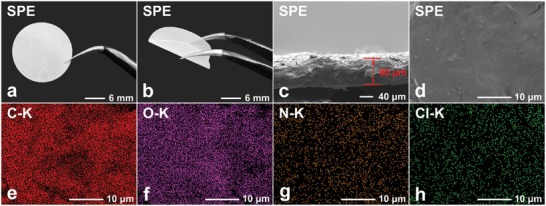
a,b) Optical photos; c,d) SEM images, and e–h) the corresponding elemental mappings of the PEO_1_‐TBMACl_1_‐SN_3_ SPE film.

**Figure** **2** and Figure S1 in the Supporting Information show the optical photos, images of scanning electron microscopy (SEM), and the corresponding elemental mapping of the PEO and PEO‐TBMACl‐SN SPE films. The PEO and PEO_1_‐TBMACl_1_‐SN_3_ samples display similar optical morphology with integrated structure and good flexibility. A much smoother surface was obtained for the PEO_1_‐TBMACl_1_‐SN_3_ SPE film (Figure S1b, Supporting Information), which has a thickness of about 80 µm (Figure 2c) and a uniform distribution of C, O, N, and Cl elements, indicating that the SN and TBMACl are well dispersed in the PEO matrix. With the increase of the SN content (PEO_1_‐TBMACl_1_‐SN_4_), the as‐prepared SPE film lost its structural integrity and many holes were formed in the film (Figure S1c,h,i, Supporting Information). This was caused by volatilization of excess SN in the ternary SPE film during the vacuum drying.

**Figure 3:**
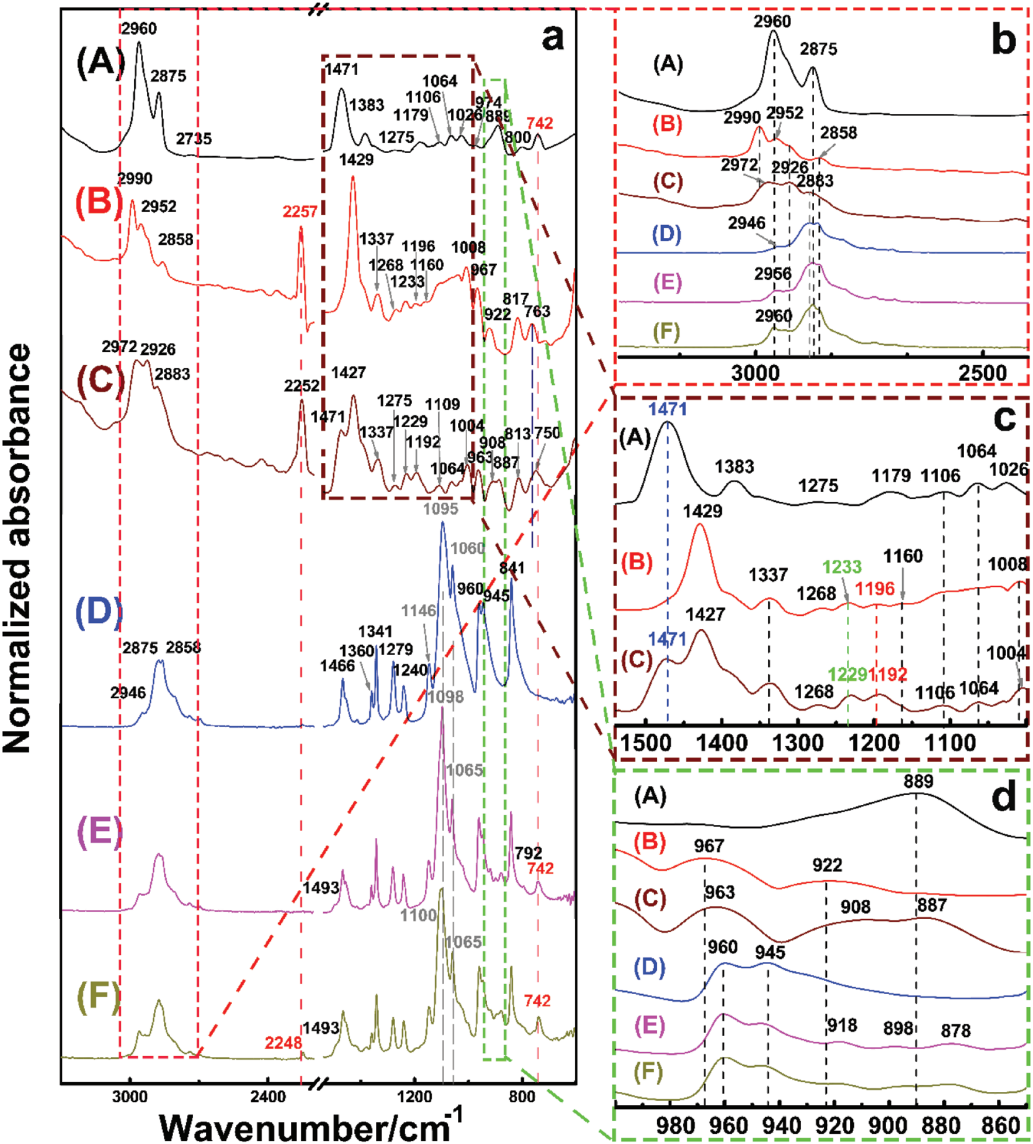
a) FTIR spectra of (A) pure TBMACl, (B) pure SN, (C) TBMACl_1_‐SN_3_, (D) pure PEO film, (E) PEO_1_‐TBMACl_1_ SPE film, and (F) PEO_1_‐TBMACl_1_‐SN_3_ SPE film. b–d) The enlarged view from (a).

Fourier transform infrared spectroscopy (FTIR) was carried out to further elucidate the interaction of the PEO, TBMACl, and SN components in the SPE films, as shown in **Figure** **3**. The characteristic assignments for these pure components are listed in Tables S1–S3 in the Supporting Information. The TBMACl_1_‐SN_3_ mixture exhibits its biphasic nature due to appearance of bands belonging to both TBMACl and SN. However, evident peak shifting was observed. The bands at 2990 and 2952 cm^−1^, which are related to CH_2_ stretching vibration of SN,^47^ move to lower wavenumbers (red‐shift) for more than 15 cm^−1^ after the addition of TBMACl. The bands corresponding to CH_2_ twsiting and rocking vibrations show a similar shifting. Meanwhile, a loss of electrons for the —C≡N occurs,^48^ leading to a red‐shift of the cyanide peak from 2257 to 2252 cm^−1^. The bands of C—CN stretching at 967 and 922 cm^−1^ also shift to lower wavenumbers. This may be ascribed to the solvation of TBMACl by SN through the interactions of —C≡N/quaternary ammonium cation (TBMA^+^) and −CH_2_/chloride ion, which are beneficial to dissociate the TBMACl. Similar solvation process has been reported in the mixtures of tetramethylammonium chloride/acetonitrile (ACN) and tetrabutylammonium chloide/acetonitrile.^49^ For the TBMACl in the SN‐TBMACl SPE film, the band at 2875 cm^−1^ related to CH_3_ symmetric streching vibration moves to a higher wavenumber for 8 cm^−1^,^50^ and the symmetric streching vibration peak of C—C—C—N at 1179 cm^−1^ shifts to a higher wavenumber of 13 cm^−1^.^51^ The strong chemical interaction between TBMACl and SN from the result of XRD is confirmed. For the binary PEO‐TBMACl SPE film, although most of the absorption peaks of TBMACl were overlapped with those of PEO, some distinct variations can still be observed. The bands assigned to the CH_2_ stretching vibration at 2960 and 1471 cm^−1^ shift to lower wavenumbers for about 4 cm^−1^.^50^ The asymmetric bending vibration peak of CH_3_ at 1471 cm^−1^ moves to a higher wavenumber for 22 cm^−1^ with a drastic decrease in peak intensity. The C—C—C symmetric streching peak at 800 cm^−1^ appears at a lower wavenumber of 792 cm^−1^ in the binary SPE film.^52^ The broad peak corresponding to the CH_2_ and CH_3_ rocking vibration at 889 cm^−1^ disappearred,^51^ and instead, new peaks at 878, 898, and 918 cm^−1^ were formed in the binary SPE film, in which the width of the band ascribed to the C—O—C stretching tripliet of PEO at 1046, 1095, and 1060 cm^−1^ was decreased,^53^ and the peak at 1060 cm^−1^ showed a sharp reduced intensity. The intensity of the CH_2_ symmetric rocking peak of PEO at 945 cm^−1^ was also weakened.^53^ In addition, the peak in the range of 2840–2925 cm^−1^ of the CH_2_ stretching of PEO was narrowed.^53^, ^54^ These changes confirm the corrdination of the quaternary ammonium cation with the ether group of PEO in the binary PEO‐TBMACl SPE film, which contributes the dissociation of TBMACl. For the binary PEO‐SN system, a systematic study reported previouly proposed that the complexation between PEO and SN occurred via the formation of hydrogen bonding between the CH_2_ of SN and the oxygen of the PEO,^45^ which will compete with chloride ion in the interaction with the CH_2_ of SN. The ternay PEO‐TBMACl‐SN SPE film shows a similar FTIR pattern with the binary PEO‐TBMACl SPE film. However, several changes can be noted. A futher decrease of the width of the CH_2_ stretching peak at 2840–2925 cm^−1^ was observed. The C—O—C stretching peak at around 1100 cm^−1^ and the CH_2_ twisting peak at 1279 cm^−1^ were further weakened. The cyanide peak of SN at 2257 cm^−1^ was significanly reduced, associated with a further red‐shift to 2248 cm^−1^. Accordingly, both TBMACl and SN are well coordinated in the PEO matrix. The solvation interactions between the TBMA^+^ cation and unshared electron pairs of the —C≡N in SN and the ether group in PEO dissociate the TBMA^+^‐Cl^−^ ion pairs and decrease the crystallinity of the ternary SPE. Furthermore, the CH_2_ of SN prefers to coordinate with the ether group in PEO, which not only further reduces the crystallinity of PEO, but also induces the decrease of the interaction between chloride ion and the CH_2_ of SN. As a result, the chloride ions interact weakly with their environment, and high chloride ion mobility between the PEO chains with enhanced free volume by the above interactions is anticipated. In contrast, the migration of the large TBMA^+^ cation along and between PEO chains is constrained by its significant interaction with the polymer chains.

**Figure 4:**
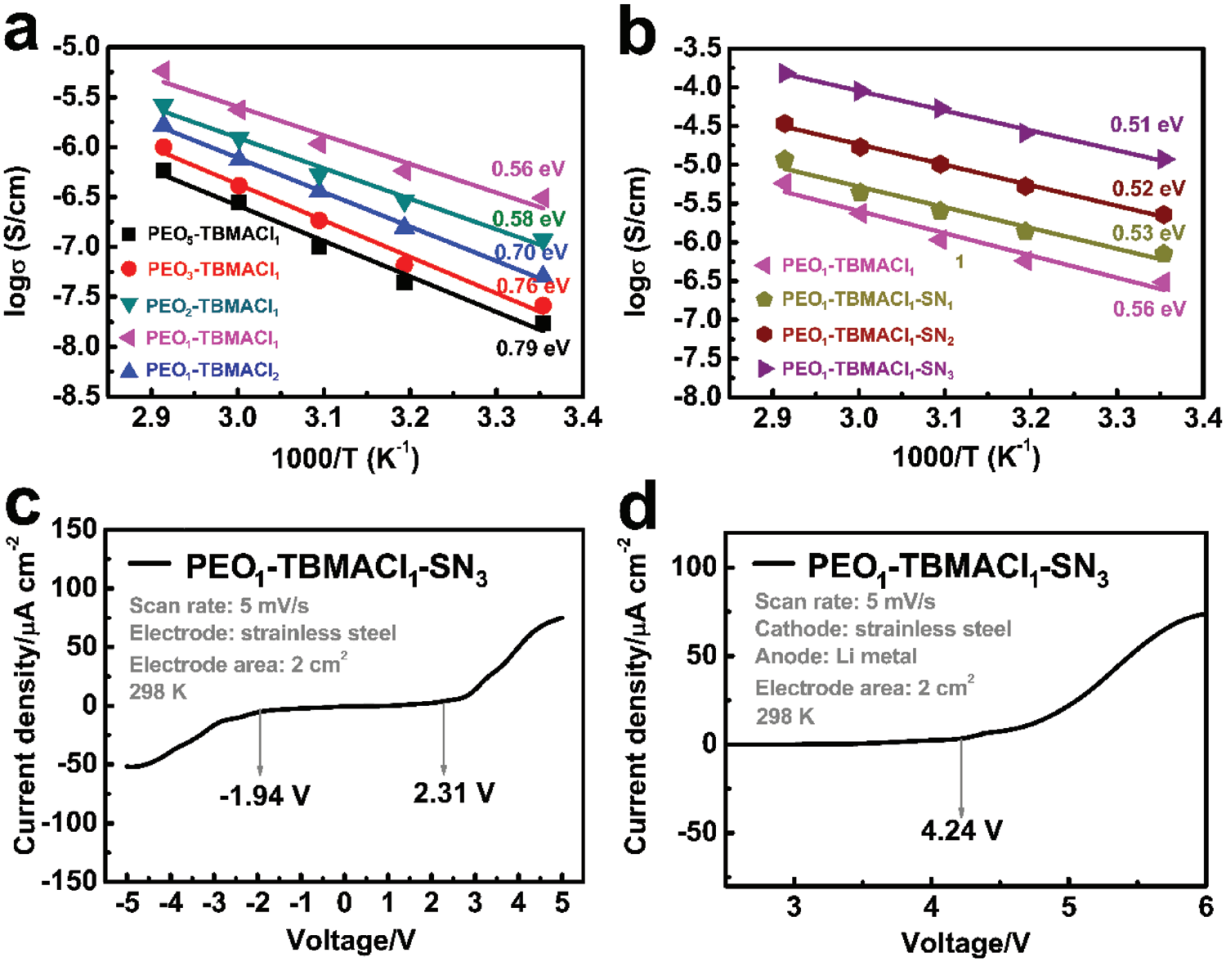
a,b) Arrhenius plots of the binary PEO‐TBMACl and ternary PEO‐TBMACl‐SN SPEs with different mass ratios. c,d) LSV scan (5 mV s^−1^) of the PEO_1_‐TBMACl_1_‐SN_3_ SPE at 298 K.

The ionic conductivity was measured from impedance measurements with two stainless steel (SS) blocking electrodes at different temperatures. Figure S2 in the Supporting Information shows the Nyquist plots of the binary PEO‐TBMACl and ternary PEO‐TBMACl‐SN SPEs at various temperatures. The spectra are composed of a depressed semicircle at high frequencies due to ion conduction and a straight line at low frequencies due to electrode polarization. The typical equivalent circuit consisting of a parallel R‐CPE circuit (the semicircles) with the bulk resistance *R* and a constant phase element CPE_1_, in series with the CPE_2_ (the straight lines), fits the measured spectra well. The ionic conductivity σ values of the electrolytes can be calculated by using the equation of σ = *L*/(*S* × *R*), where L is the membrane thickness and S is the effective electrode area, as listed in Table S4 in the Supporting Information. For all the binary and ternary samples, the ionic conductivity increases linearly with the temperature. The temperature dependence of the electrolytes can be described by an Arrhenius‐like behavior. **Figure** **4** shows the Arrhenius plots for the ionic conductivities of the PEO‐TBMACl and PEO‐TBMACl‐SN SPEs. The PEO_5_‐TBMACl_1_ SPE shows a very low concuctivity of 1.7 × 10^−8^ S cm^−1^ at 298 K. The conductivity of this binary SPE system increases with the increase of the TBMACl concentration, which provides more charge carriers for transport. Meanwhile, the crystallinity of the PEO‐TBMACl decreases, as indicated by the evident decrease of the melting enthalpy from 174.0 (PEO) to 134.4 J g^−1^ (PEO_1_‐TBMACl_1_) derived from the DSC result, facilitating the ion transport. The conductivity reaches a maximum value of 3.1 × 10^−7^ S cm^−1^ at 50% TBMACl (PEO_1_‐TBMACl_1_), that is, ≈20 times the value of the PEO_5_‐TBMACl_1_ SPE, followed by a decrease with further increase of the TBMACl content (PEO_1_‐TBMACl_2_). This decrease is ascribed to the “inactive” pure TBMACl that segregated from the PEO matrix, as shown in the XRD pattern (Figure 1a), blocking the conducting pathways.

With the addition of SN at low concentration in the binary PEO_1_‐TBMACl_1_ SPE, there was a mild increase of the ionic conductivity from 3.1 × 10^−7^ (PEO_1_‐TBMACl_1_) to 6.7 × 10^−7^ S cm^−1^ (PEO_1_‐TBMACl_1_‐SN_1_), although a large melting enthalpy change from 134.4 to 103.2 J g^−1^ was obtained. This may indicate that the SN at low concentration in the ternary SPE plays a dominant role in the decrease in crystallinity (i.e., the increase of ion transport pathways), instead of the dissociation of TBMACl. When a high concentration of SN was added (PEO_1_‐TBMACl_1_‐SN_3_), a remarkable enhancement of the ionic conductivity to 1.2 × 10^−5^ S cm^−1^, which is about 40 times of 3.1 × 10^−7^ (PEO_1_‐TBMACl_1_), together with a medium variation of the melting enthalpy from 103.2 to 84.2 J g^−1^, is received. This conductivity enhancement mainly comes from the further effective dissociation of TBMACl by SN, associated with an increase of the amorphous fraction, promoting the ion transport in the SPE.

The further increase of the SN content in the ternary SPE led to the formation of inactive SN, which would be volatilized during the vacuum drying and consequently brought about the formation of inhomogeneous film (Figure S1, Supporting Information). The activation energies calculated from the Arrhenius plots in the temperature range from 298 to 333 K are also marked in Figure 4. The activation energy was drasticly decreased from 0.79 to 0.56 eV with the increase of the TBMACl content in the binary PEO‐TBMACl system. A continious decrease of the activation energy was observed with the additon of SN and a lower value of 0.51 eV for the PEO_1_‐TBMACl_1_‐SN_3_ SPE was obtained.

Besides the ionic conductivity, the electrochemical window (EW) is also a determining factor for achieving high performance CIBs. The linear sweep voltammetry (LSV) was measured for the as‐prepared PEO_1_‐TBMACl_1_‐SN_3_ SPE using the SS/SS and SS/Li electrode couples, as shown in Figure 4c,d and Figure S3 in the Supporting Information. By using the SS/SS electrode couple, the as‐prepared PEO_1_‐TBMACl_1_‐SN_3_ SPE shows the anodic and cathodic potentials of 2.31 and −1.94 V (vs SS), respectively, i.e., an electrochemical window of 4.25 V at 298 K. The anodic stability of the as‐prepared PEO_1_‐TBMACl_1_‐SN_3_ SPE has also been evaluated in a SS/PEO_1_‐TBMACl_1_‐SN_3_/Li cell. A high value of 4.24 V relative to lithium metal was obtained at 298 K. This means that the cut‐off voltage for the charging process of CIBs with lithium metal as anode could be set at 4.24 V, which provides a stable environment for the electrochemical reactions in metal chlorides/metal, metal oxychlorides/metal, and chloride ion–doped conducting polymer/metal electrode systems that have been proposed for CIBs. Moreover, this value is higher than those of the liquid electrolytes such as the binary ionic liquids (4.0 V) and the mixture of chloride ionic liquid/propylene carbonate (3.2 V),^16^, ^24^, ^29^ suggesting a high electrochemical stability of the as‐prepared PEO_1_‐TBMACl_1_‐SN_3_ SPE. The electrochemical stability is almost unchanged with the increase of the temperature to 323 K (Figure S3, Supporting Information).

**Figure 5:**
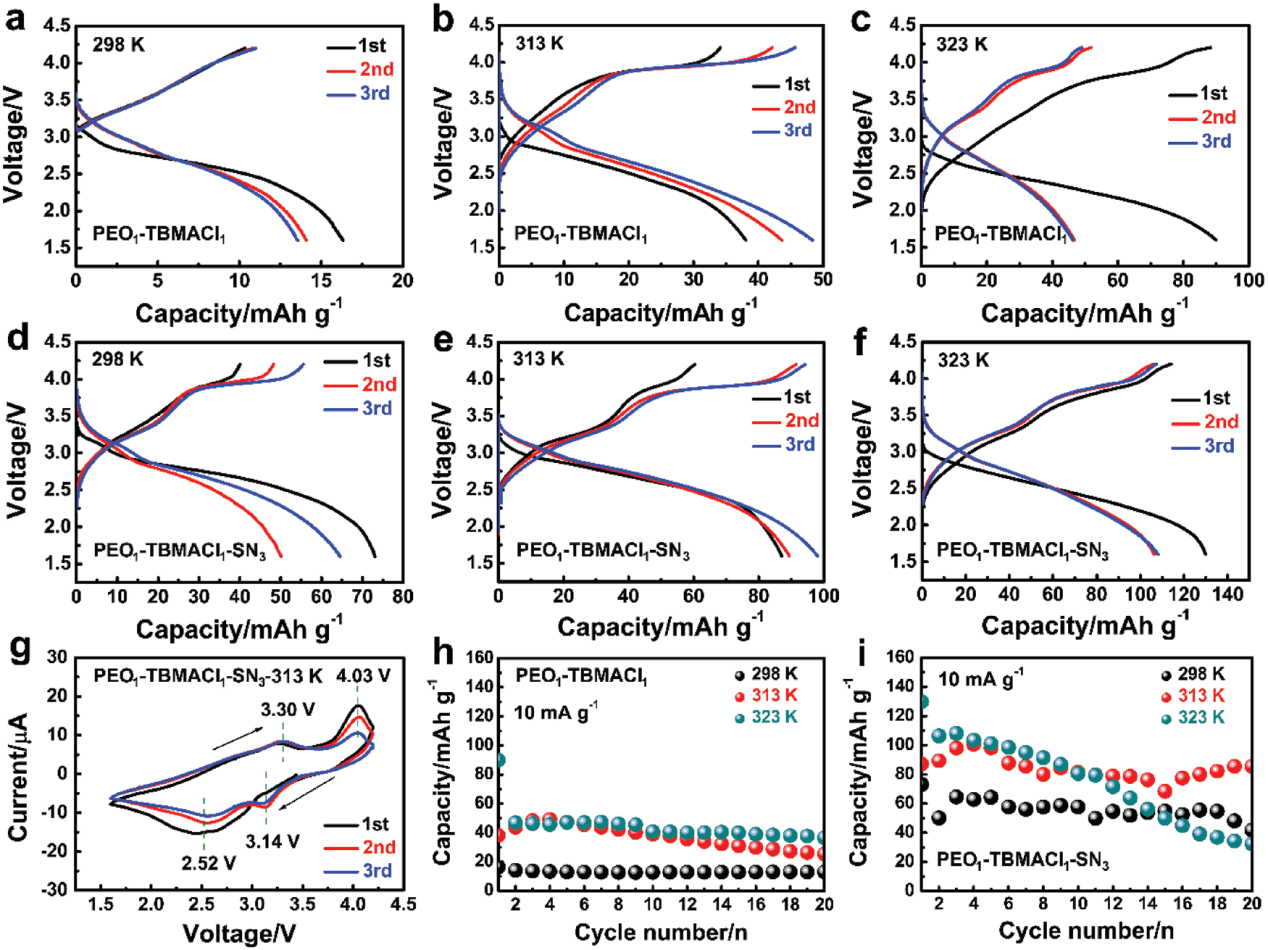
Discharge and charge curves and cycling performance of the FeOCl/Li batteries using a–c,h) PEO_1_‐TBMACl_1_ or d–f,i) PEO_1_‐TBMACl_1_‐SN_3_ SPE at 298, 313, and 323 K. g) CV patterns (50 µV s^−1^) of the FeOCl/Li battery using the PEO_1_‐TBMACl_1_‐SN_3_ SPE at 313 K.

All‐solid‐state batteries were fabricated using the as‐prepared binary PEO_1_‐TBMACl_1_ or ternary PEO_1_‐TBMACl_1_‐SN_3_ film as the electrolyte, which was sandwiched between the FeOCl cathode and the lithium metal anode. **Figure** **5** shows the electrochemical properties of the all‐solid‐state batteries at different temperatures. All the batteries exhibit an increase of the discharge capacity as the operating temperature increases, which is consistent with the variation of the ionic conductivity of the SPEs as a function of the temperature. The battery using the binary PEO_1_‐TBMACl_1_ SPE shows a very low initial discharge capacity, which is calculated based on the active material of FeOCl cathode, of 18 mAh g^−1^, which can be increased to more than 40 mAh g^−1^ at the elevated temperatures. However, a large voltage gap between discharge and charge of about 1.25 V still exists. When the ternary PEO_1_‐TBMACl_1_‐SN_3_ SPE is used, a much higher discharge capacity of 73 mAh g^−1^ is obtained at 298 K. A further enhancement in discharge capacity (87 mAh g^−1^), as well as the reversiblity of discharge and charge, is received as the operating temperature increases to 313 K (Figure 5e). Moreover, this battery displays distinct discharge and charge pleateaus and a significantly reduced voltage hysteresis to about 0.8 V. Two discharge and charge stages can be distinguished. This is in accordance with the result of cyclic voltammetry (CV) patterns (Figure 5g), which exhibit two pairs of cathodic and anodic peaks after the first activation under the potential window of 1.6–4.2 V. The first reduction peak appears at 3.14 V and a subsequent reduction peak is centered at 2.52 V. The corresponding oxidation peaks are located at 4.03 and 3.30 V, respectively. By employing a higher operating temperature of 323 K, the reversible capacity in the initial cycles is increased to about 110 mAh g^−1^ with a high Coulombic efficiency of about 99%. The cycling test for the batteries is presented in Figure 5h,i. All the batteries using the PEO_1_‐TBMACl_1_ or ternary PEO_1_‐TBMACl_1_‐SN_3_ SPE have good cycling stability at 298 and 313 K, demonstrating the high electrochemical stability of the as‐prepared SPEs in the large voltage range. An evident capacity decay of the FeOCl/PEO_1_‐TBMACl_1_‐SN_3_/Li battery is observed after the initial cycles at 323 K. This may be ascribed to interruption of mass transfer between the FeOCl cathode and the SPE by the large volume change of the FeOCl at a high capacity. Actually, the capacity decay of the FeOCl cathode also occurred in the liquid electrolyte of CIBs. It can be reduced by decreasing the charge and discharge capacity, that is, the reduction of volume change, as shown in Figure S4 in the Supporting Information. Therefore, the development of an appropriate technology for the electrode preparation of the all‐solid‐state battery is required for realizing superior cycling stability at a high capacity.

To further understand the reasons for the better battery performance by using the ternary PEO_1_‐TBMACl_1_‐SN_3_ SPE as compared with the binary PEO_1_‐TBMACl_1_ SPE, electrochemical impedance spectroscopy (EIS) measurement was performed (Figure S5, Supporting Information). The EIS curves exhibit a semicircle at high frequency followed by a further semicircle and a straight line at low frequency, indicating a mixed rate‐determining electrochemical process containing charge transfer and ion diffusion. The former semicircle is related to the interface resistance, the latter semicircle is considered to be the charge transfer resistance, and the straight line is associated with the Warburg impedance by ion diffusion. The measured EIS plots can be fitted well using the equivalent circuit in Figure S5b in the Supporting Information. It is clear that both the interface resistance and charge transfer resistance, as well as the Warburg impedance, are significantly reduced using the ternary PEO_1_‐TBMACl_1_‐SN_3_ SPE, indicating its superior electrochemical properties.

**Figure 6:**
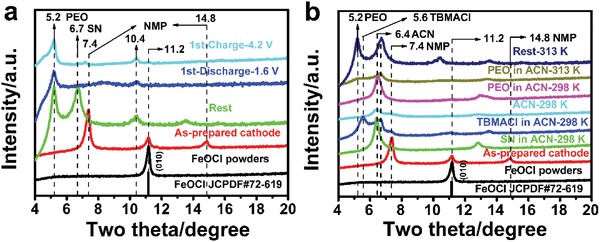
a) XRD patterns of the as‐prepared FeOCl powders and the as‐prepared FeOCl cathodes at the rest, discharge, or charge state in the first cycle. b) XRD patterns of the as‐prepared FeOCl powders and the as‐prepared FeOCl cathodes treated by ACN, SN/ACN, TBMACl/ACN, or PEO/ACN at 298 or 313 K for 8 h.

In order to reveal the electrochemical reaction mechanism of the all‐solid‐state battery using the ternary PEO_1_‐TBMACl_1_‐SN_3_ SPE, microstructural characterizations were carried out. **Figure** **6** and Figure S6 in the Supporting Information show the ex situ XRD patterns of the FeOCl cathodes at various electrochemical and chemical states. The diffraction peaks of the as‐prepared FeOCl powders can be well indexed and assigned to the orthorhombic FeOCl phase (PDF card no. 72‐619). The FeOCl interlayer is reflected by the main diffraction peak at 11.2° (i.e., 0.79 nm of the interlayer spacing), which was markedly weakened, and a new diffraction peak at about 7.4° (1.19 nm) was formed after the electrode preparation by the slurry coating method. This irreversible lattice expansion has been proved to be caused by the intercalation of *N*‐methyl‐2‐pyrrolidinone (NMP),^26^ which was removed after the electrode drying under vacuum. Upon the rest of the coin cell at 313 K before the discharge and charge testing, both the pristine peak at 11.2° and the NMP intercalation induced peak at 7.4° of the FeOCl cathode disappeared, and instead, another two new peaks at lower positions of 6.7° (1.37 nm) and 5.2° (1.70 nm) appeared. This means that the FeOCl interlayer spacing along the *b*‐axis direction was greatly expanded before cycling. Our previous theoretical and experimental results suggested that both organic molecule (NMP or acetonitrile) and ionic compound in the electrolyte (1‐butyl‐1‐methylpiperidinium chloride) could be readily intercalated into the FeOCl interlayer at room temperature.^26^, ^55^ Therefore, the intercalation behavior of each component of the as‐prepared PEO_1_‐TBMACl_1_‐SN_3_ SPE in the FeOCl interlayers was investigated through the immersion test in the acetonitrile solutions (Figure 6b). The immersion of the as‐prepared FeOCl electrode into the pure ACN or SN/ACN solution results in a fully intercalation of FeOCl with a reflection peak at 6.4° (1.39 nm). When the as‐prepared FeOCl electrode was immersed in the TBMACl/ACN solution, the peak corresponding to the intercalation of ACN slightly shifted to a higher degree of 6.7°, and a new peak at 5.6° (1.57 nm) was formed, which may be attributed to the intercalation of TBMACl. The immersion test was also performed in the PEO/ACN solution. Only the intercalation of ACN was observed at 298 K. When the temperature increased to 313 K, the new peak at 5.2° was formed, reflecting a large interlayer spacing of 1.70 nm of FeOCl. This is likely due to the intercalation of PEO. Actually, the intercalation of the large molecule of PEO, together with cations of lithium ions or protons, in the layered materials such as the expanded MoS_2_ or titanate has been previously reported.^56^, ^57^ For the FeOCl material, the intercalation of the small molecule SN and the ionic compound TMBACl is reasonable due to the high affinity of −CH_2_ and TBMA^+^ with the chloride ion in the interlayers of FeOCl. The intercalation of PEO is also possible because of the aforementioned strong coordination interactions among SN, TMBACl, and PEO, which bind them together and help the PEO to access into the interlayers of FeOCl.

**Figure 7:**
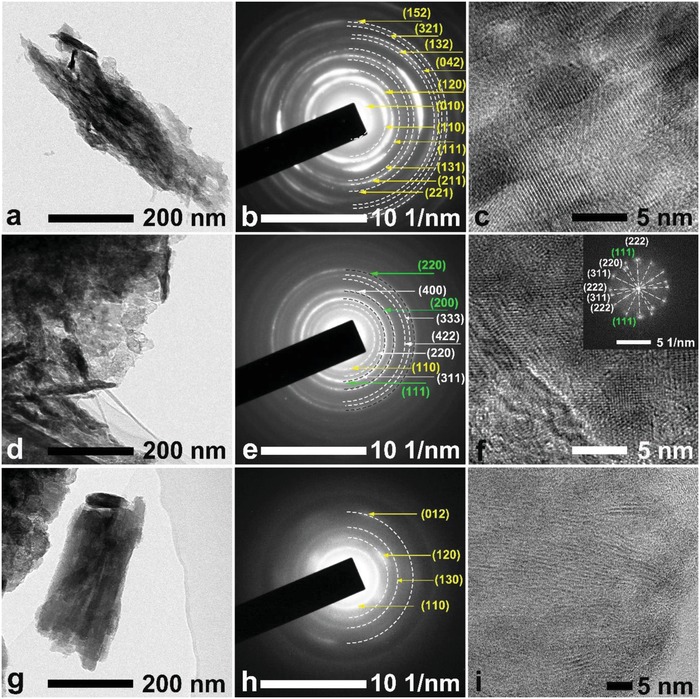
TEM, SAED, HRTEM, and FFT patterns of the FeOCl cathodes at different electrochemical states in the first cycle: a–c) as‐prepared; d–f) fully discharged; and g–i) fully charged. The inset in the panel (f) is the corresponding FFT pattern.

After the first discharge to 1.6 V at 313 K, the diffraction peak of the FeOCl cathode at 6.7° vanished and other peaks with evidently decreased intensity still exist. Some of the intercalated FeOCl phase did not participate in the electrochemical reaction. This is consistent with the result of the discharge that only 87 mAh g^−1^ was delivered, which is much lower than the theoretical capacity (249.7 mAh g^−1^) of FeOCl. The following charge to 4.2 V did not change the peak position, but increased the peak intensity, accompanied by the peak broadening. A weak peak at 11.2° was also observed. This indicates a recovery of the FeOCl species with a reduced grain size. The energy‐dispersive spectroscopy (EDS) analysis for the FeOCl cathodes washed with ACN in Figure S7 in the Supporting Information demonstrated the decrease and recovery of the chlorine content during discharge and the subsequent charge, respectively. This suggests the chloride ion transfer occurs at the FeOCl cathode side during cycling. The transmission electron microscopy (TEM) analysis was implemented to gain an insight into the phase transformation of the FeOCl cathode in the first cycle. **Figure** **7** shows the TEM images, the corresponding selected area electron diffraction (SAED) patterns, and high‐resolution TEM (HRTEM) images of the FeOCl cathodes. The distinct crystalline structure of the as‐prepared FeOCl material is confirmed by the SAED result (Figure 7b). Upon discharge, the iron oxides of cubic FeO and Fe_3_O_4_ phases with poor crystallinity are formed, as reflected by the characteristic lattice planes of (111), (200), and (220) for FeO (PDF card no. 6‐615), and (220), (311), (400), and (422) for Fe_3_O_4_ (PDF card no. 75‐1372). The HRTEM and the corresponding fast Fourier transform (FFT) results (Figure 7f) confirmed the formation of the nanocrystalline iron oxides containing ferrous ion after the discharge of FeOCl. The formation of Fe_3_O_4_ could be ascribed to the oxidation of the discharge product of FeO during the TEM test. Similar phase transformation was reported in our previous work using the liquid electrolyte.^25^, ^26^ In the following charge, as indexed in the SAED pattern (Figure 7h), the corresponding nanocrystalline FeOCl phase, which is different from the pristine crystalline FeOCl, was formed and showed a grain size of less than 10 nm (Figure 7i). These findings prove that the chloride ion transfer and the redox reaction of iron species are involved in the reversible electrochemical reaction of the as‐prepared FeOCl cathode.

**Figure 8:**
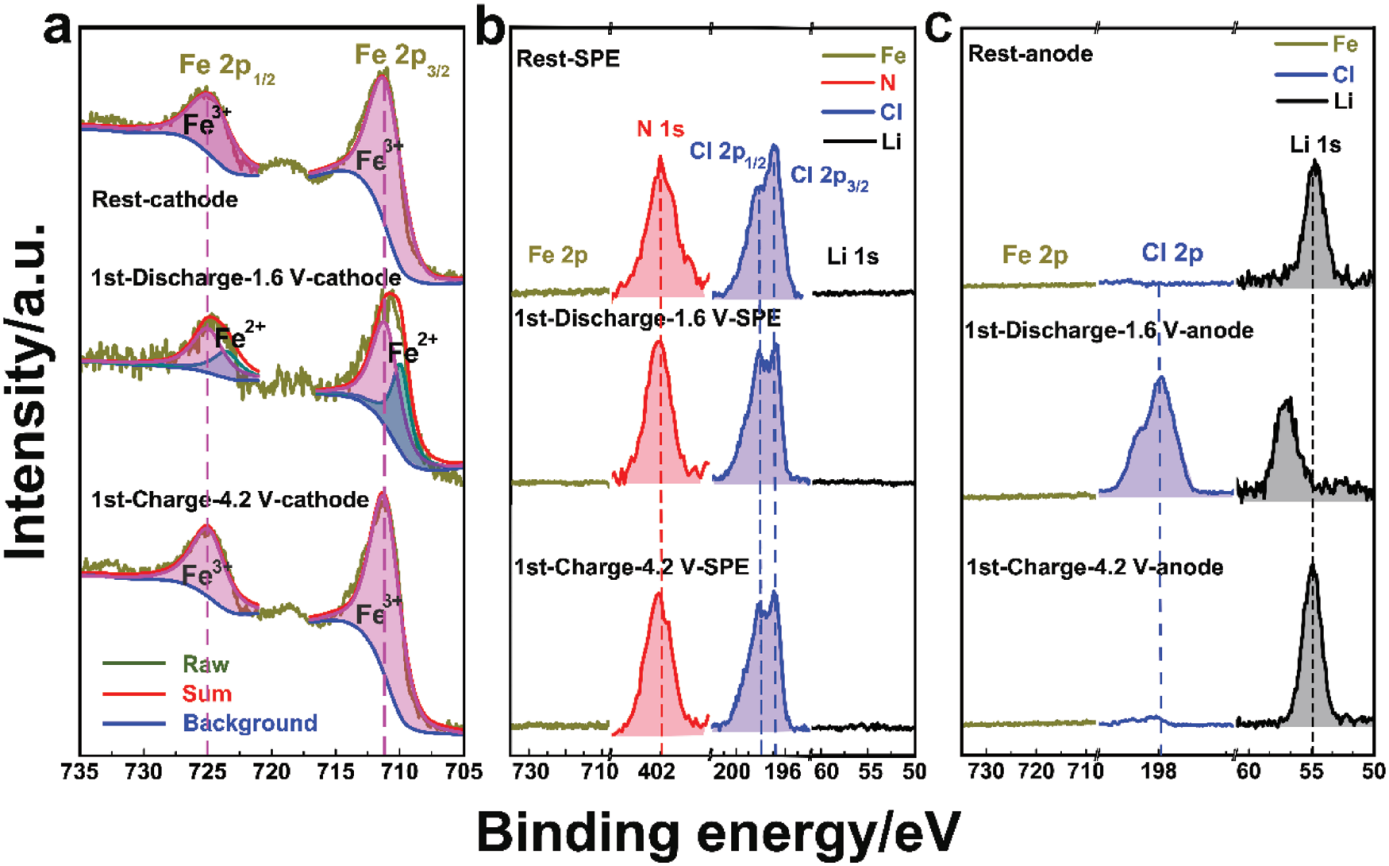
XPS region spectra of a) the FeOCl cathodes; b) the PEO_1_‐TBMACl_1_‐SN_3_ SPE; and c) the Li anode at the rest, fully discharged, and fully charged states in the first cycle.

**Figure 9:**
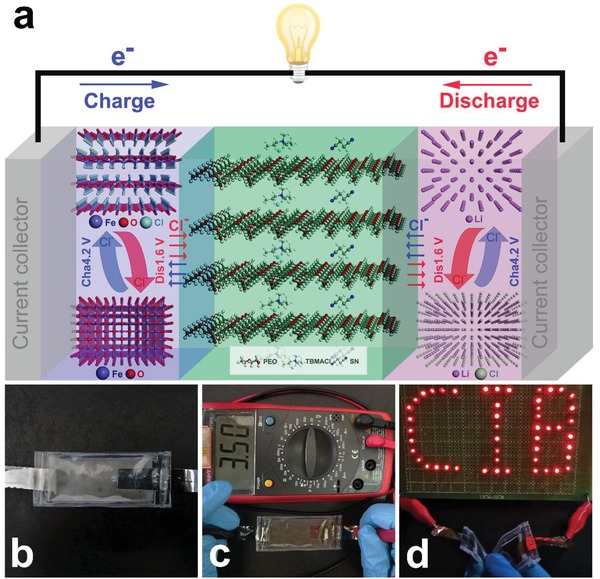
a) Schematic diagram of the ASS‐RCIB with the PEO_1_‐TBMACl_1_‐SN_3_ SPE and the FeOCl/Li electrode couple. b) Optical photos of a soft‐packing ASS‐RCIB. c) Open circuit potential of flexible soft packet ASS‐RCIB. d) Optical image of red LEDs powered by the soft‐packing ASS‐RCIB.

Although chloride ion transfer is involved in both the FeOCl cathode and the SPE, lithium ion transfer in the all‐solid‐battery is not precluded as the lithium metal was used as the anode, in which the electrochemical reaction mechanism also needs to be clarified. Therefore, further investigation was performed. **Figure** **8** and Figures S8 and S9 in the Supporting Information show the X‐ray photoelectron spectroscopy (XPS) analysis for the FeOCl cathode, PEO_1_‐TBMACl_1_‐SN_3_ SPE, and lithium anode of the all‐solid‐state battery. From the XPS survey spectra (Figure S8, Supporting Information), it is clear that the iron species exist only at the cathode side and the lithium species are found only at the anode side regardless of the different electrochemical states, suggesting the high stability between the electrodes and the SPE, and the cation transfer is not included in the battery. More detailed and definite information is provided in the XPS region spectra (Figure 8). The as‐prepared FeOCl cathode after the rest process before cycling shows its Fe 2p peaks at 711.4 (Fe 2p_3/2_) and 725.1 eV (Fe 2p_1/2_), and Cl 2p peaks at 198.8 (Cl 2p_3/2_) and 200.4 eV (Cl 2p_1/2_). Those values are identical to the binding energies (BEs) of the Fe 2p and Cl 2p of the as‐prepared FeOCl powders (Figure S8d and Figure S9, Supporting Information), indicating that the phase structure of the FeOCl was not changed by the abovementioned chemical intercalations. Upon discharge, new Fe 2p peak doublet located at lower binding energies of 709.8 and 723.5 eV was found and can be assigned to Fe^2+^ species (FeO), which confirms the TEM result. In the following charge, the Fe 2p signals belonging to the FeOCl phase were recovered. The corresponding chlorine content in the FeOCl cathode decreased after discharge and then increased by the recharge (Figure S8d, Supporting Information). These observations support the above reaction mechanism proposed for the FeOCl cathode. For the region spectra of the PEO_1_‐TBMACl_1_‐SN_3_ SPE (Figure 8b), there is no evident variation of the N 1s, Cl 2p_3/2_, and Cl 2p_1/2_ characteristic signals before and after cycling, which lie at about 402.0, 196.9, and 198.4 eV, respectively. Moreover, the Fe and Li species are definitely not included in the SPEs during cycling according to the absence of the Fe 2p and Li 1s signals. For the lithium anode, the Li 1s peak of the pristine sample is centered at 54.8 eV, which could be assigned to lithium metal, and also LiOH and/or Li_2_CO_3_ because of the oxidation of the extremely sensitive Li in air during the sample loading for the XPS test. After the discharge, the shifting of the Li 1s peak to a higher binding energy of 56.6 eV, together with the emergence of the Cl 2p peak doublet at 197.9 (Cl 2p_3/2_) and 199.5 eV (Cl 2p_1/2_), occurred, providing the proof for the formation of lithium chloride. This discharge product was reduced to the pristine lithium state, as indicated by the return of the Li 1s peak. The EDS analysis for all the components in the all‐solid‐state battery, as shown in Figures S10–S12 in the Supporting Information, was also investigated, and the results showed that the chlorine element appeared at the Li anode side after discharge and disappeared upon the following charge. Furthermore, the iron element exists only at the cathode side. As demonstrated by the above observations and analysis, we propose the chloride ion transfer reaction mechanism for the FeOCl/ PEO_1_‐TBMACl_1_‐SN_3_/Li all‐solid‐state battery, namely, the all‐solid‐state rechargeable chloride ion battery, in which redox reactions of FeOCl/FeO and Li/LiCl occurred at the cathode and anode, respectively. The as‐prepared SPE balances the battery reactions by allowing the chloride ion shuttle between the cathode and anode, as illustrated in **Figure** **9**a. A soft‐packing ASS‐RCIB was assembled (Figure 9b), showing an open circuit potential of 3.5 V (Figure 9c) and good flexibility. It can readily power multiple commercial red light‐emitting diodes (LEDs), as shown in Figure 9d, demonstrating the first practical ASS‐RCIB using a solid polymer electrolyte.

## 3 Conclusion

In summary, we developed the first all‐solid‐state rechargeable chloride ion battery by employing a ternary solid polymer electrolyte, which is composed of a PEO polymer matrix, a quaternary ammonium chloride salt, and a solid SN plasticizer. The as‐prepared SPE shows conductivities of 10^−5^–10^−4^ S cm^−1^ in the temperature range of 298–343 K and a high electrochemical stability with the anodic potential of more than 4.2 V versus Li. The chemical interactions, as confirmed by XRD, DSC, and FTIR characterizations, among components of the SPE contributed to the dissociation of the chloride salt and thus bring about the notable increase of the conductivity, resulting in the evident increase of the reversible capacity of the ASS‐RCIB. The electrochemical reaction mechanism of the ASS‐RCIB using the SPE is based on the chloride ion shuttle via the redox reactions of FeOCl/FeO at the cathode side and Li/LiCl at the anode side, as validated by XRD, TEM, XPS and electrochemical measurements.

## 4 Experimental Section

PEO with an average molecular weight of 300 000 g mol^−1^, SN (99%), anhydrous acetonitrile (99.8% and packaged under argon), and anhydrous NMP (99.5% and packaged under argon) were purchased from Alfa Aesar. TBMACl (99%) was obtained from Lanzhou Institute of Chemical Physics. PEO and TBMACl were vacuum dried at 313 and 353 K for 48 h before use, respectively. The SPE films were prepared by a solution casting method. For the binary PEO‐TBMACl series, the PEO and TBMACl with a mass ratio range from 5:1 to 1:2 were used. The optimized PEO‐TBMACl composition with a mass ratio of 1:1 was selected to prepare the ternary PEO‐TBMACl‐SN series. The PEO and TBMACl were dissolved in ACN, followed by the addition of different amounts of SN. The mixture was stirred for 48 h at 450 rpm to obtain a homogeneous viscous solution, which was casted onto Teflon plate and allowed to evaporate the ACN solvent in a nitrogen‐filled box for 48 h. Afterward, the samples were dried under vacuum at 323 K for 48 h and stored in an argon‐filled glove box. The schematic illustration for preparation of the SPE films is shown in Scheme S1 in the Supporting Information. The synthesis of the FeOCl cathode material was performed by a thermal decomposition of FeCl_3_·6H_2_O at 493 K for 1 h. Then, the product was thoroughly washed with acetone, followed by an overnight drying under vacuum at 333 K.

Powder XRD patterns were collected on a Rigaku SmartLab diffractometer using Cu Kα radiation. SEM/EDS (Ultra55) and TEM/EDS results were obtained on the Field‐emission Ultra55 and Tecnai G2 F20 U‐TWIN microscopes, respectively. The samples were washed with anhydrous NMP and ACN to remove the poly(vinylidene difluoride) (PVDF) and the SPE in the glove box before the TEM test. DSC and TGA thermograms were acquired at a ramp rate of 10 °C min^−1^ under a nitrogen atmosphere using the NETZSCH STA449F1instrument. FTIR spectra over the range of 400–4000 cm^−1^ were received using a Thermo Nicolet Nexus 670 FT‐IR spectrometer. XPS was recorded by an AXIS Ultra DLD spectrometer with Al Kα excitation. The C 1s line with a BE of 284.8 eV was used as a standard.

LSV testing for the SPEs in the coin cells was carried out at the scan of 5 mV s^−1^ using the SS/SS and SS/Li electrode couples at different temperatures on the Bio‐Logic (VMP3) electrochemical workstation, which was also employed to collect the EIS (100 kHz to 1 Hz) data of the SPEs in the Swagelok cells containing two polished SS blocking electrodes in the temperature range of 298–343 K. The battery testing was performed using the FeOCl cathode, SPE, and lithium metal (China energy lithium Co. Ltd.) anode in coin cells. The FeOCl cathode was fabricated by mixing the as‐prepared FeOCl powders, PVDF, and carbon black in the mass ratio of 60:10:30 and a subsequent slurry coating with graphite foil (17 µm, Suzhou Dasen Electronics Material Co. Ltd.) as the current collector. Discharge and charge testing was implemented galvanostatically at 10 mA g^−1^ over a voltage range between 1.6 and 4.2 V by using Neware battery testing system (Neware Co. Ltd.) at 298, 313, or 323 K. The specific capacities were calculated according to the mass of the as‐prepared FeOCl powders in the electrodes. EIS (100 kHz to 10 mHz) data and CV (1.6 to 4.2 V, 50 µV s^−1^) of the batteries using the FeOCl working electrode were all collected under a Bio‐Logic (VMP3) electrochemical workstation.

## Conflict of Interest

The authors declare no conflict of interest.

## Supporting information

Supplementary
