# Supplementary material for: An All‐Solid‐State Rechargeable Chloride Ion Battery
Source: Adv Sci (Weinh). 2019 Jan 28;6(6):1802130. doi: 10.1002/advs.201802130 (PMC6425448; doi:10.1002/advs.201802130)
Supplement: Supplementary file 1 — Supplementary [file ADVS-6-1802130-s001.pdf]

Copyright WILEY-VCH Verlag GmbH & Co. KGaA, 69469 Weinheim, Germany, 2019.

ADVANCED SCIENCE
Open Access

Supporting Information

for *Adv. Sci.,* DOI: 10.1002/advs.201802130

An All-Solid-State Rechargeable Chloride Ion Battery

Chao Chen, Tingting Yu, Meng Yang, Xiangyu Zhao, and Xiaodong Shen*

Copyright WILEY-VCH Verlag GmbH & Co. KGaA, 69469 Weinheim, Germany, 2018.

Supporting Information

An all-solid-state rechargeable chloride ion battery

Chao Chen, Tingting Yu, Meng Yang, Xiangyu Zhao*, Xiaodong Shen

C. Chen, Dr. M. Yang, Prof. X. Y. Zhao, Prof. X. D. Shen
College of Materials Science and Engineering
Jiangsu Collaborative Innovation Center for Advanced Inorganic Functional Composites
Nanjing Tech University, 30 Puzhu South Road, Nanjing 211816, China
Email: xiangyu.zhao@njtech.edu.cn
Prof. X. D. Shen
State Key Laboratory of Materials-Oriented Chemical Engineering
Nanjing Tech University, 30 Puzhu South Road, Nanjing 211816, China

Keywords: all-solid-state rechargeable batteries, solid electrolytes, polymer electrolytes, chloride ion batteries

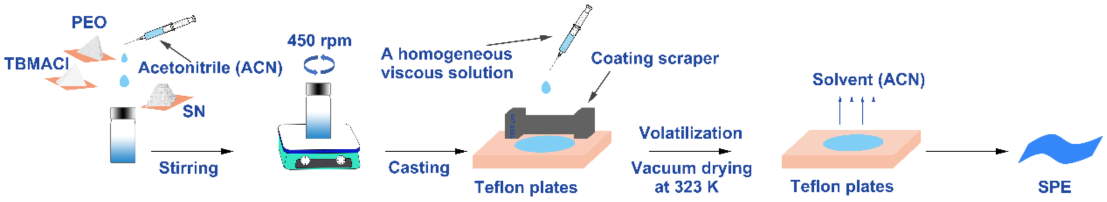

Scheme S1. The schematic illustration for preparation of the SPEs.

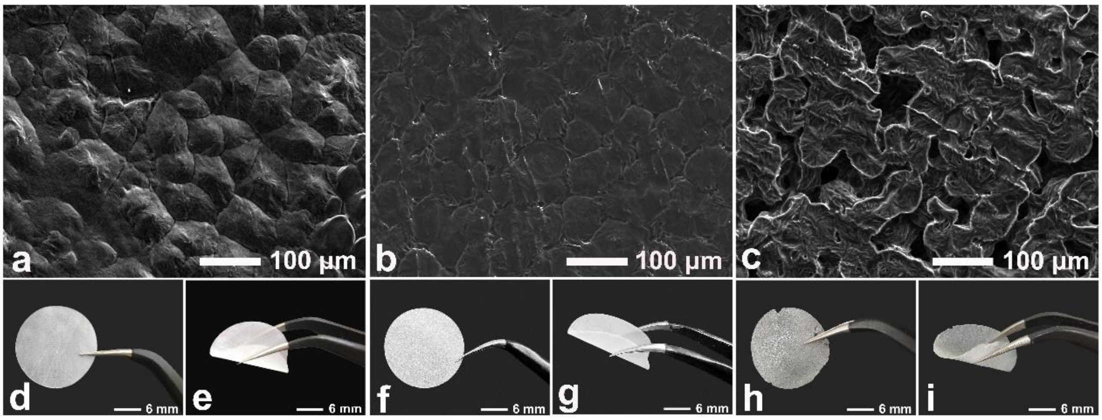

Figure S1. SEM images and the corresponding optical photos of SPE films. a,d,e) pure PEO film; b,f,g) PEO_1-$TBMACl_1$-SN_3 SPE film; and c,h,j) PEO_1-$TBMACl_1$-SN_4 SPE film.

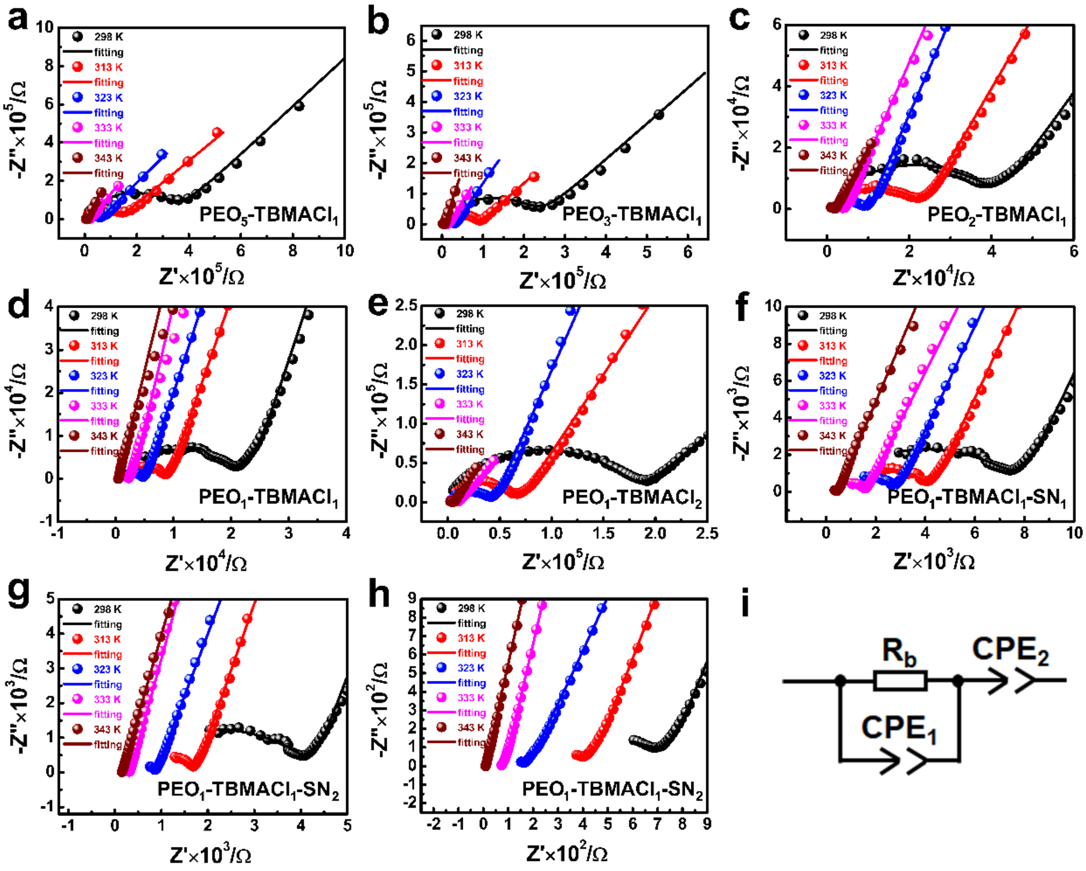

Figure S2. a-h) Nyquist plots of the binary PEO-TBMACl and ternary PEO-TBMACl-SN SPEs at various temperatures. i) the equivalent circuit for the impedance plots of the SPEs.

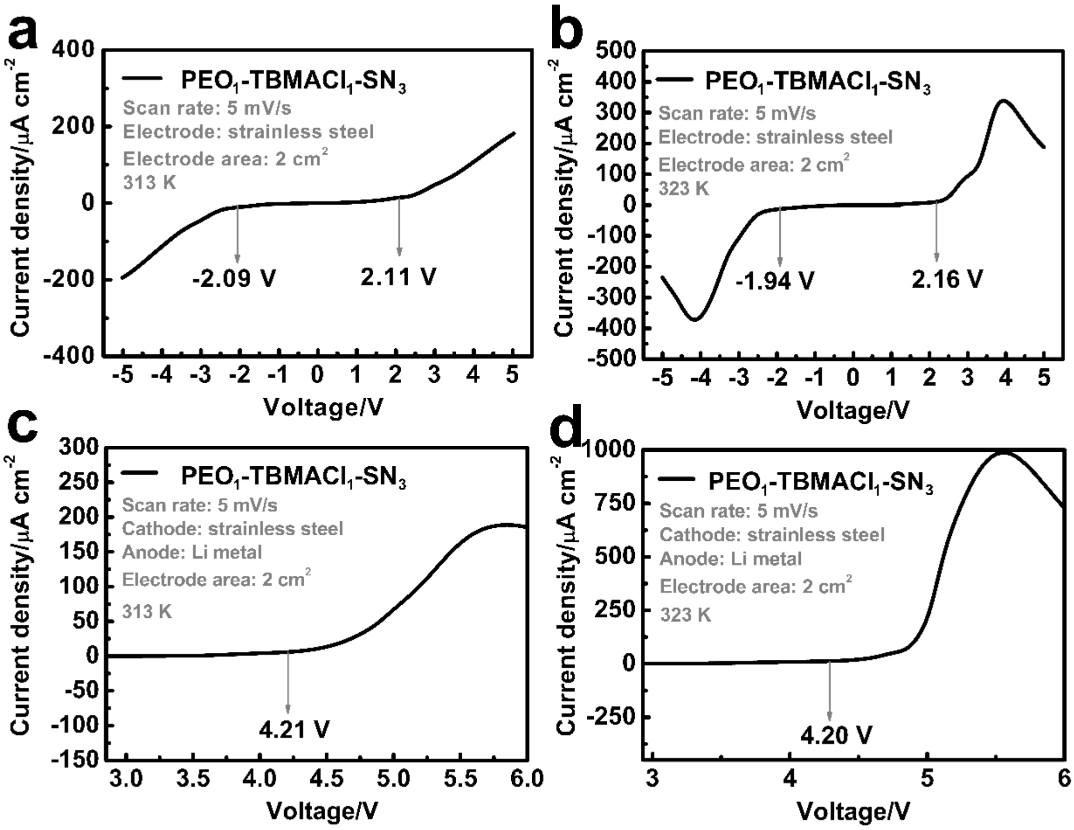

Figure S3. LSV scans (5 mV s^{-1}) of the PEO_1-$TBMACl_1$-SN_3 SPEs: a,c) 313 K; b,d) 323 K.

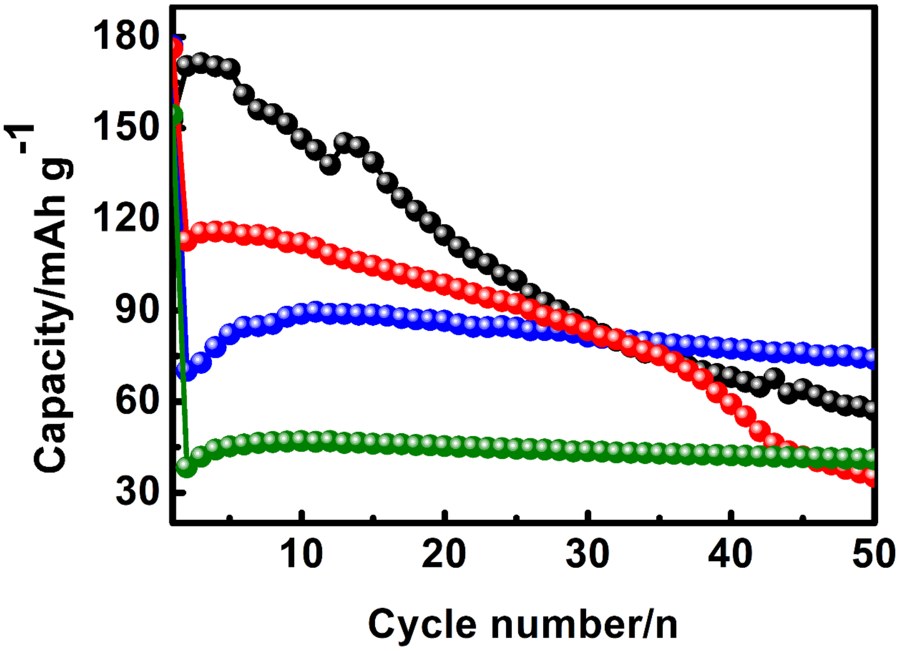

Figure S4. Cycling performance of the FeOCl cathodes at different discharge terms in the liquid electrolyte (0.5 M $PP_{14}Cl$ in $PP_{14}TFSI$). The FeOCl powders were prepared by a thermal decomposition of $FeCl_3·6H_2O$. The FeOCl cathode was fabricated by mixing the as-prepared FeOCl powders, PVDF and carbon black in the mass ratio of 60:10:30 and a subsequent slurry coating with graphite foil. Discharge and charge testing of the FeOCl cathodes were implemented galvanostatically (10 mA g^{-1}) at different discharge cut-off voltages (i.e., different theoretical discharge capacities and volume changes): greed solid circle, 2.5 V; blue solid circle, 2.2 V; red solid circle, 2.1 V; black solid circle, 1.6 V. It can be clearly seen that the FeOCl shows a severe capacity decay when a higher discharge capacity was delivered. A superior cycling performance was received at a lower discharge capacity. The high capacity would cause a large volume change by the phase transformation, which may interrupt the electrical contact in the cathode and thus a deterioration of cycling stability.

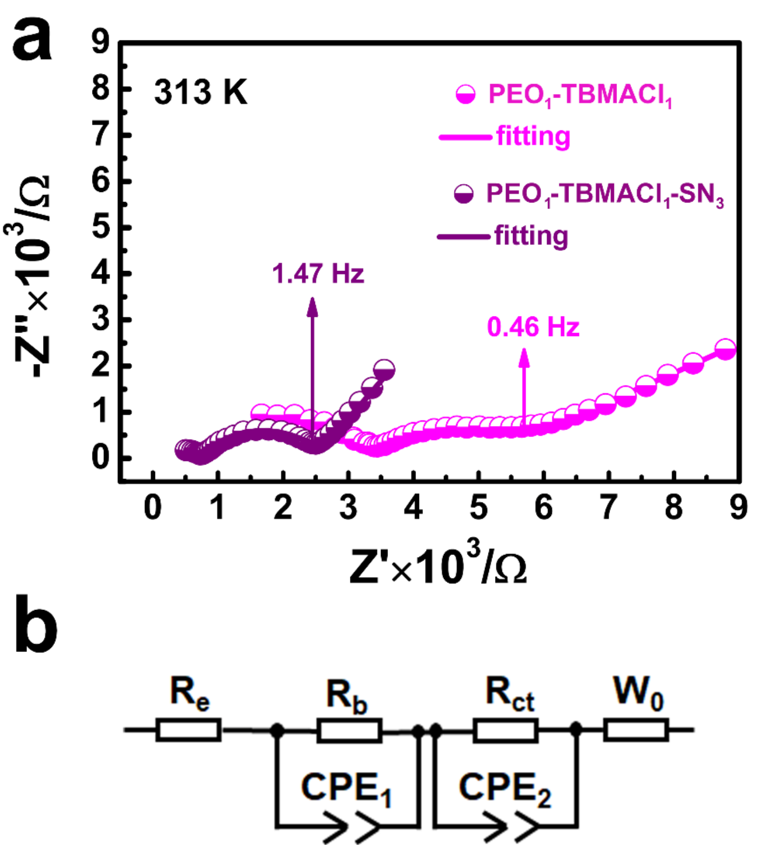

Figure S5. a) EIS patterns of the FeOCl cathodes in the battery systems using PEO_1-$TBMACl_1$ or PEO_1-$TBMACl_1$-SN_3 SPE at 313 K. b) the equivalent circuit. R_e represents the resistance arising from the cell components. R_b and CPE_1, which are associated with the medium-frequency semicircle, are the bulk resistance and its associated capacitance. R_{ct} is the charge transfer resistance and CPE_2 is the associated double-layer capacitance that represents the interfacial compatibility between the electrode and the electrolyte. W_0 is the Warburg impedance related to ion diffusion.

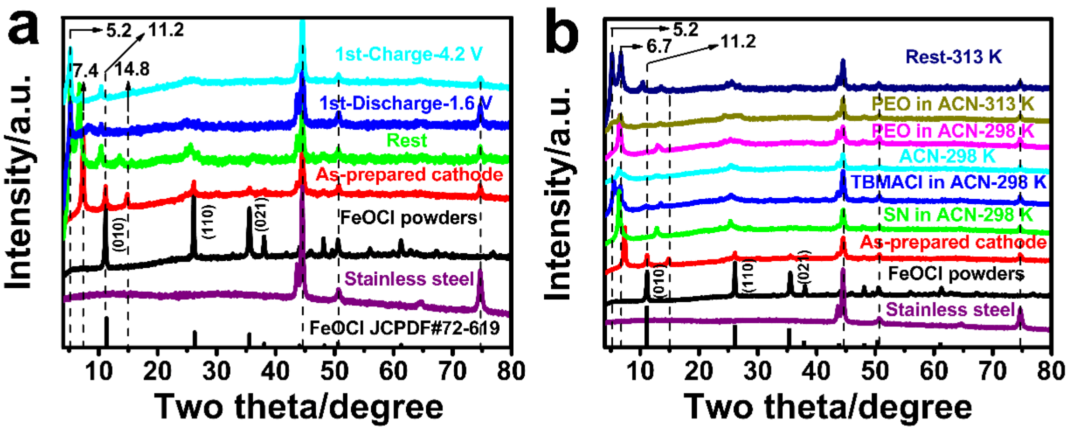

Figure S6. a) XRD patterns of the as-prepared FeOCl powders and the as-prepared FeOCl cathodes at the rest, discharge or charge state in the first cycle. b) XRD patterns of the as-prepared FeOCl powders and the as-prepared FeOCl cathodes treated by ACN, SN/ACN, TBMACl/ACN, or PEO/ACN at 298 or 313 K for 8 h.

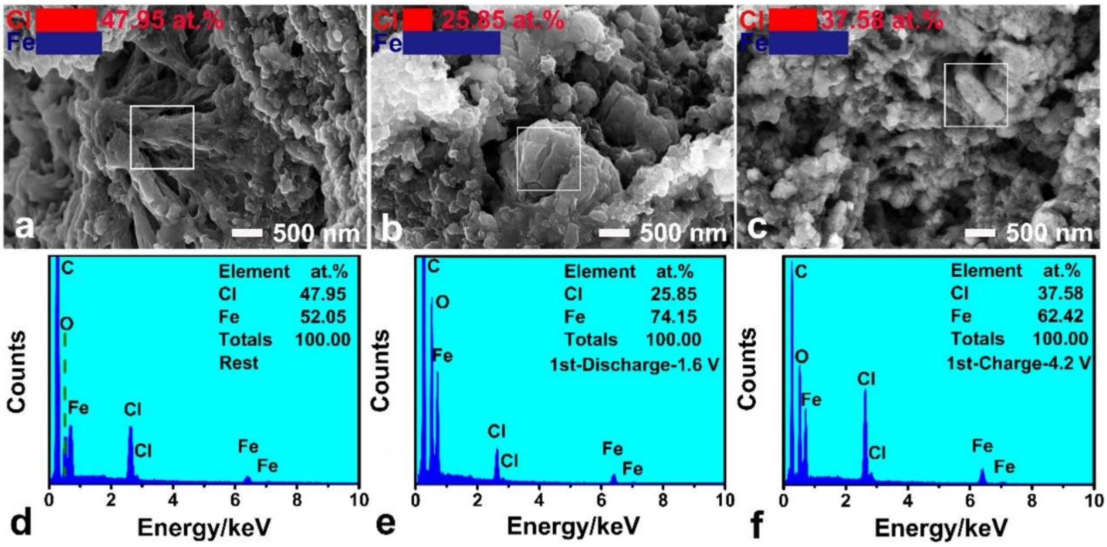

Figure S7. SEM images and the EDS patterns of the as-prepared FeOCl cathodes before and after the first cycle: a,d) rest; b,e) fully discharged; and c,f) fully charged. The white squares in a,b,c) correspond to the collecting areas of EDS.

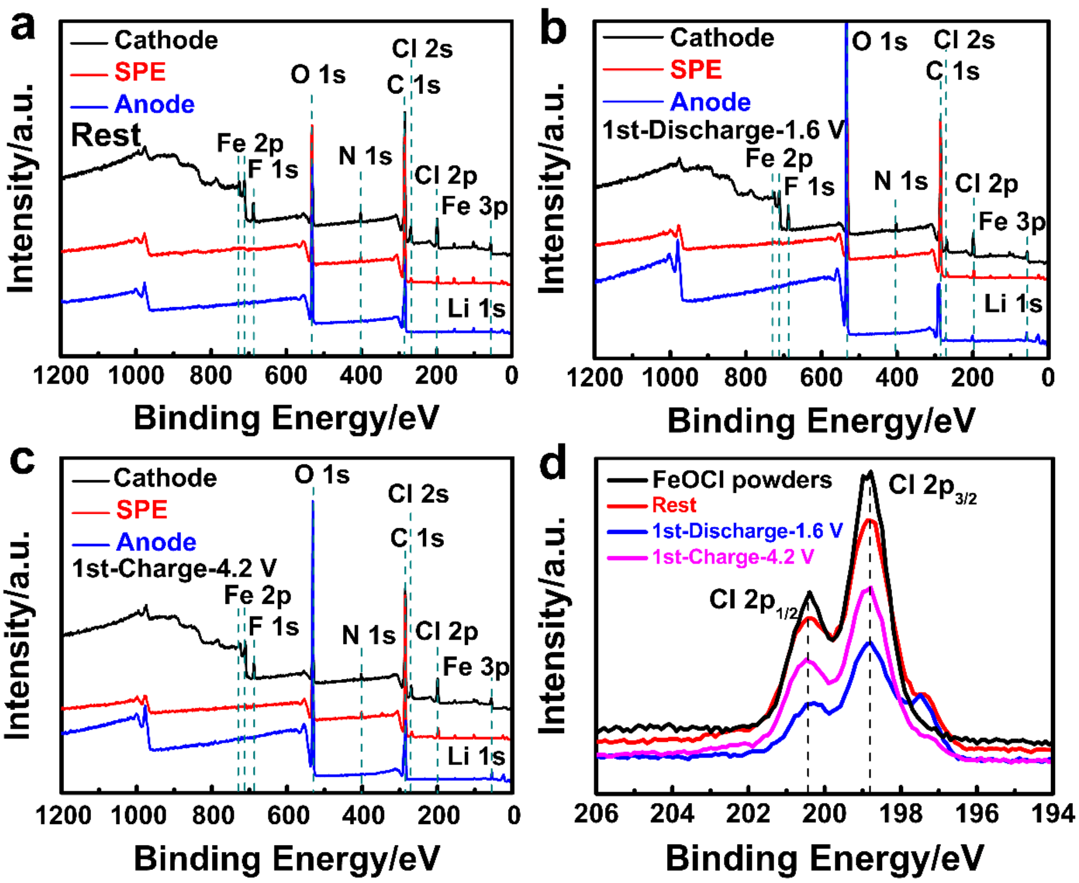

Figure S8. a-c) XPS survey spectra of the FeOCl cathodes, PEO_1-$TBMACl_1$-SN_3 SPE and the Li anode after the rest, fully discharge and fully charge in the first cycle. d) XPS region spectra of Cl 2p in the as-prepared FeOCl powders, the as-prepared FeOCl cathodes at different electrochemical states in the first cycle.

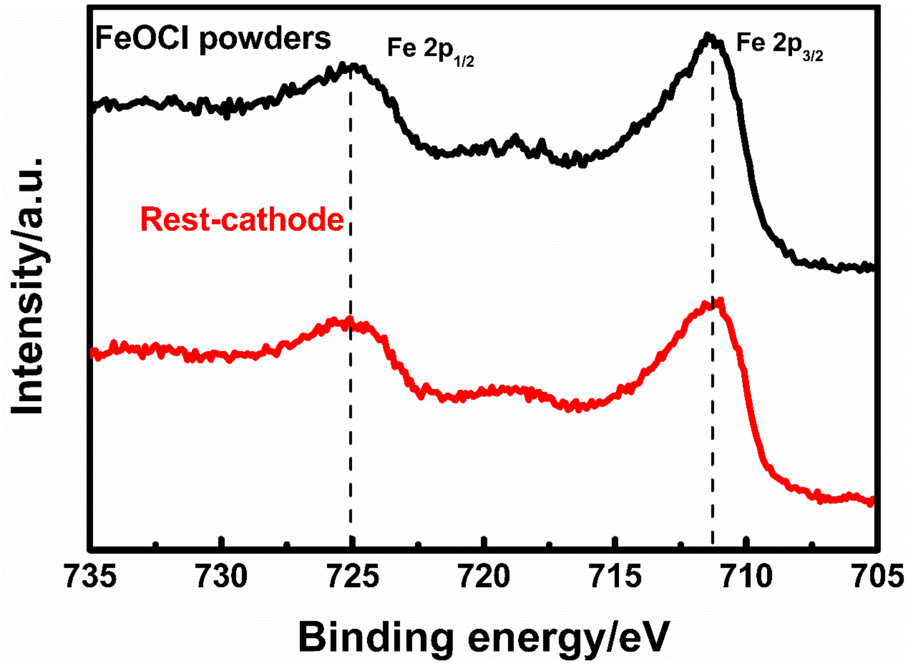

Figure S9. XPS region spectra of Fe 2p in the as-prepared FeOCl powders and the as-prepared FeOCl cathode after the rest.

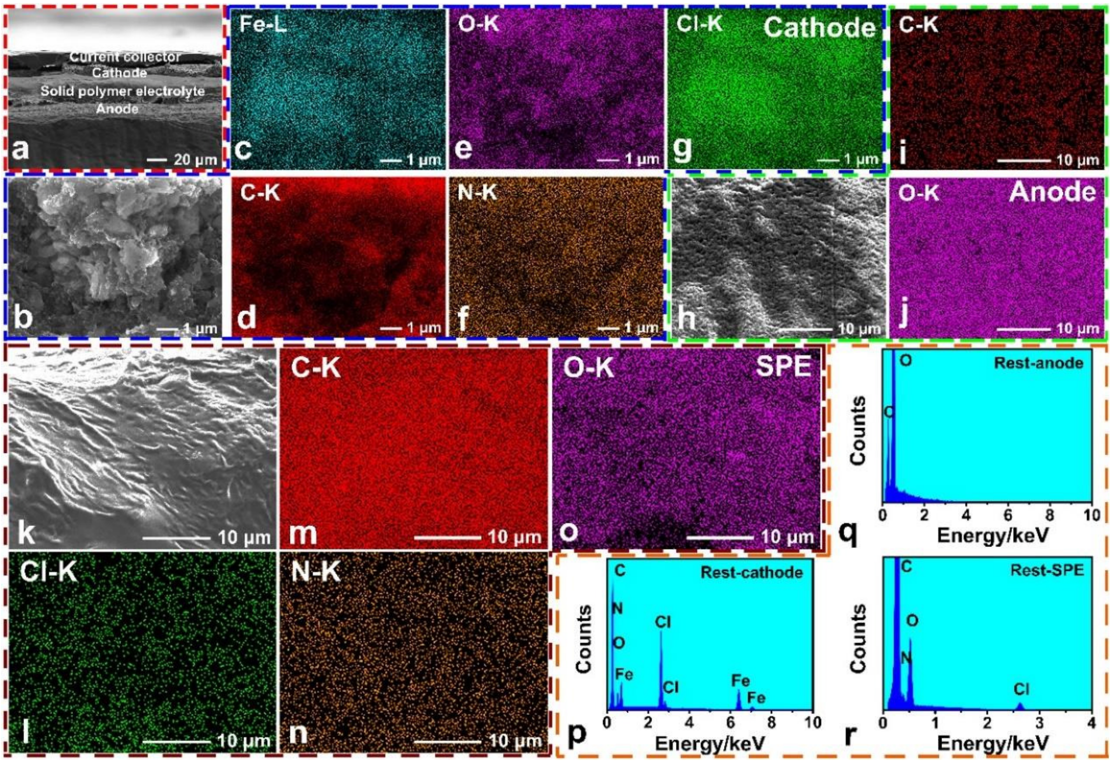

Figure S10. a) Cross-section SEM of the battery structure. SEM images and the corresponding EDS results of the FeOCl cathode, PEO_1-$TBMACl_1$-SN_3 SPE and lithium anode after the rest: b-g,p) the FeOCl cathode; h-j,q) the Li anode; k-o,r) the SPE.

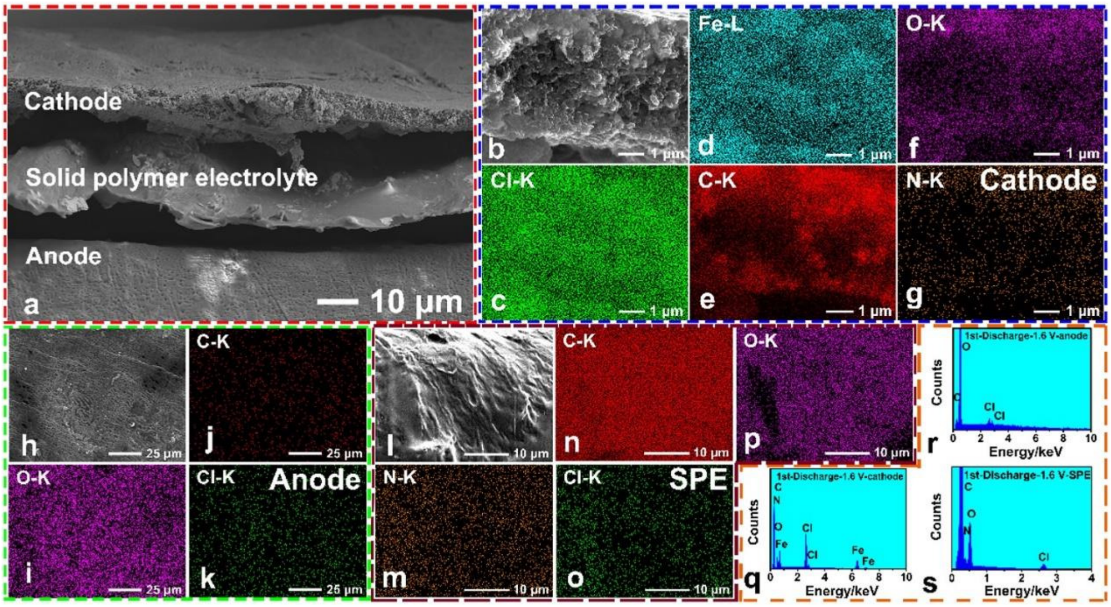

Figure S11. a) Cross-section SEM of the battery structure. SEM images and the corresponding EDS results of the FeOCl cathode, PEO_1-$TBMACl_1$-SN_3 SPE and lithium anode after the first discharge: b-g,q) the FeOCl cathode; h-k,r) the Li anode; l-p,s) the SPE.

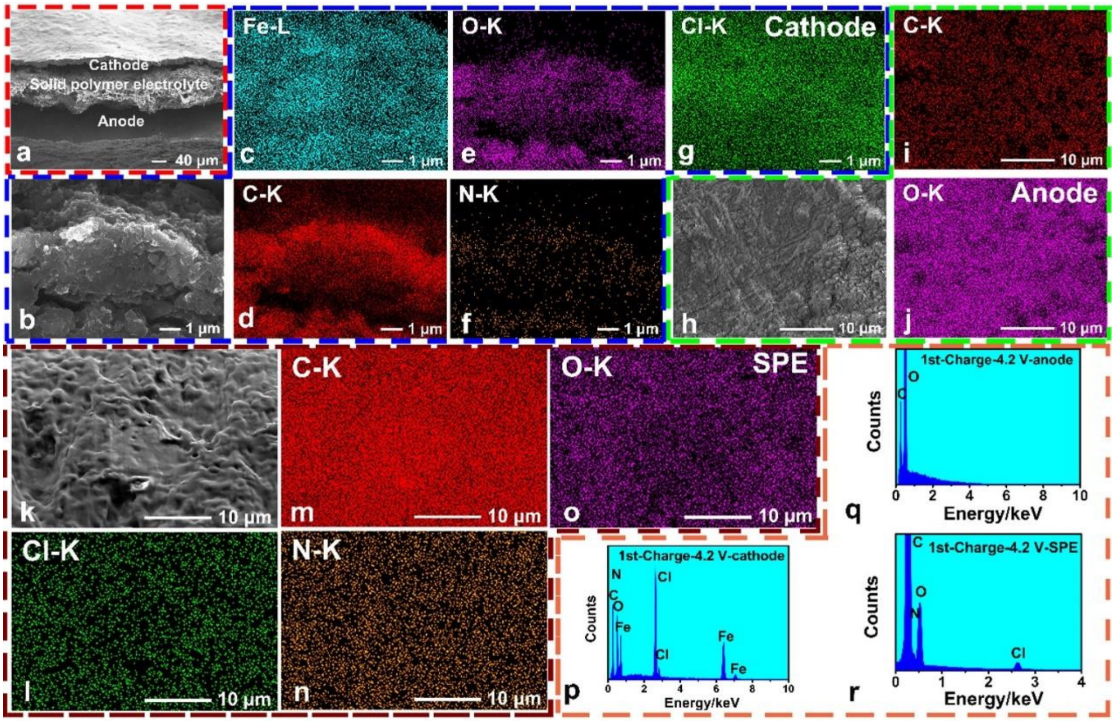

Figure S12. a) Cross-section SEM of the battery structure. SEM images and the corresponding EDS results of the FeOCl cathode, PEO_1-$TBMACl_1$-SN_3 SPE and lithium anode after the first charge: b-g,p) the FeOCl cathode; h-j,q) the Li anode; k-o,r) the SPE.

Table S1. The assignments of FTIR spectrum for PEO

Wavenumbers (cm^{-1})	Vibration mode	References
2946, 2875, 2858	CH_2 stretching	[1,2]
1466	CH_2 scissoring	[1]
1360, 1341	CH_2 wagging doublet	[1]
1279, 1240	CH_2 twisting	[1]
1146, 1095, 1060	C-O-C stretching triplet	[1]
960, 945	CH_2 symmetric rocking	[1]
841	CH_2 asymmetric rocking	[1]

Table S2. The assignments of FTIR spectrum for SN

Wavenumbers (cm^{-1})	Vibration mode	References
2990	CH_2 asymmetric stretching, gauche and trans	[3]
2952	CH_2 symmetric stretching, gauche and trans	[3]
2858	CH_2 symmetric stretching, gauche	[3]
2257	CN stretching, gauche and trans	[3]
1429, 1337	CH_2 stretching, trans	[3]
1268	CH_2 wagging, trans	[4]
1233, 1196, 1160	CH_2 twisting, gauche and trans	[5]
1008	CH_2, gauche	[3]
967	C-CN, gauche	[3]
922	C-CN, trans	[3]
817	CH_2 bending, gauche	[3]
763	CH_2 rocking, trans	[3]

Table S3. The assignments of FTIR spectrum for TBMACl

Wavenumbers (cm^{-1})	Vibration mode	References
2960, 2875, 2735	CH_3 symmetric stretching	[6]
1471	CH_3 asymmetric bending	[6]
1383	CH_3 symmetric bending	[6]
1275	CH_2 twisting	[7]
1179	C-C-C-N symmetric stretching	[8]
1106	C-C-N symmetric stretchin	[8]
1064	C-C asymmetric stretching	[8]
1026	C-C-N symmetric stretching	[8]
974	C-N symmetric stretching	[7]
889	CH_2 and CH_3 rocking	[8]
800	C-C-C symmetric stretching	[7]
742	CH_3 rocking	[6]

Table S4. The ionic conductivities of the as-prepared SPEs at different temperatures

	Ionic conductivity (S cm^{-1})				
**Samples**	298 K	313 K	323 K	333 K	343 K
**PEO_5-$TBMACl_1$**	1.7×10^{-8}	4.4×10^{-8}	1.0×10^{-7}	2.8×10^{-7}	5.9×10^{-7}
**PEO_3-$TBMACl_1$**	2.6×10^{-8}	6.6×10^{-8}	1.9×10^{-7}	4.1×10^{-7}	1.0×10^{-6}
**PEO_2-$TBMACl_1$**	1.2×10^{-7}	2.3×10^{-7}	5.3×10^{-7}	1.2×10^{-6}	2.6×10^{-6}
**PEO_1-$TBMACl_1$**	3.1×10^{-7}	5.8×10^{-7}	1.1×10^{-6}	2.4×10^{-6}	5.8×10^{-6}
**PEO_1-$TBMACl_2$**	5.0×10^{-8}	1.3×10^{-7}	2.6×10^{-7}	9.4×10^{-7}	1.7×10^{-6}
**PEO_1-$TBMACl_1$-SN_1**	6.7×10^{-7}	1.3×10^{-6}	1.9×10^{-6}	3.3×10^{-6}	1.1×10^{-5}
**PEO_1-$TBMACl_1$-SN_2**	2.1×10^{-6}	5.1×10^{-6}	8.0×10^{-6}	2.0×10^{-5}	4.0×10^{-5}
**PEO_1-$TBMACl_1$-SN_3**	1.2×10^{-5}	2.6×10^{-5}	5.3×10^{-5}	8.9×10^{-5}	1.5×10^{-4}

References

[1] Y. L. Ni'mah, M-Y. Cheng, J. H. Cheng, J. Rick, B-J. Hwang, J. Power Sources **2015**, 278, 375.

[2] R. K. Gupta, H. W. Rhee, B. Korean Chem. S. **2017**, 38, 356.

[3] D. Zhou, Y. He, R. Liu, M. Liu, H. D. Du, B. H. Li, Q. Cai, Q-H. Yang, F. Y. Kang, Adv. Energy Mater. **2015**, 5, 1500353.

[4] S. Das, A. J. Bhattacharyya, Solid State Ionics **2010**, 181, 1732.

[5] R. K. Gupta, H. W. Rhee, Electrochim. Acta **2012**, 76, 159.

[6] M. Karbowiak, J. Hanuza, J. Janczak, J. Drozdzynski, J. Alloys Compd. **1995**, 225, 338.

[7] J. Tarasiewicz, R. Jakubas, J. Baran, A. Pietraszko, J. Mol. Struct. **2004**, 697, 161.

[8] A. Oueslati, F. Hlel, M. Gargouri, Ionics **2010**, 17, 91.
